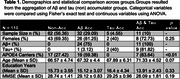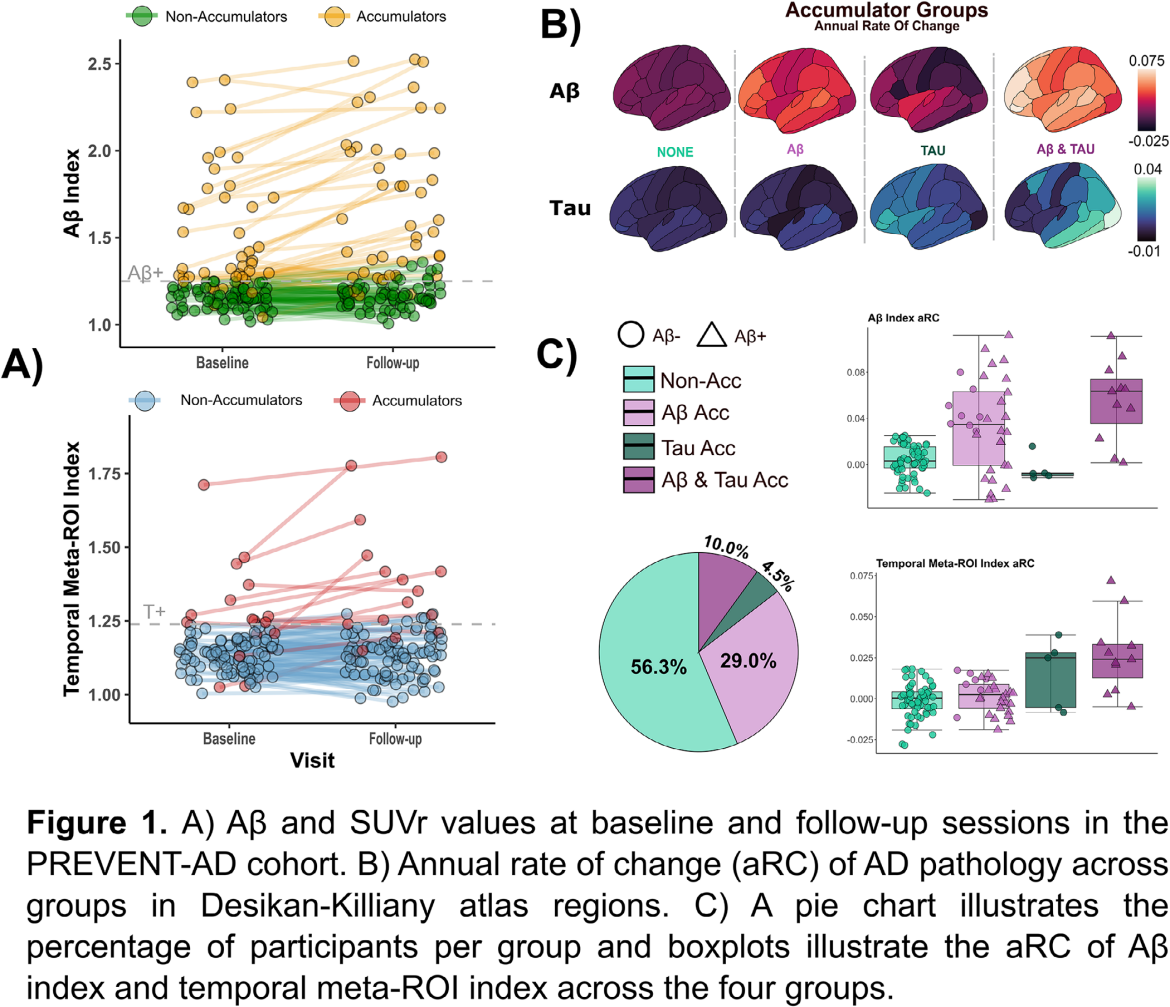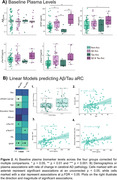# Longitudinal change of cerebral amyloid and tau and its association with plasma biomarkers in preclinical Alzheimer's disease

**DOI:** 10.1002/alz.093070

**Published:** 2025-01-09

**Authors:** Alfonso Fajardo, Yara Yakoub, Jonathan Gallego Rudolf, Jordana Remz, Jean‐Paul Soucy, Nicholas J. Ashton, Henrik Zetterberg, Kaj Blennow, John C.S. Breitner, Judes Poirier, Sylvia Villeneuve, PREVENT‐AD Research Group

**Affiliations:** ^1^ Douglas Mental Health University Institute, Centre for Studies on the Prevention of Alzheimer's Disease (StoP‐AD), Montréal, QC Canada; ^2^ Douglas Mental Health Research Centre, Montreal, QC Canada; ^3^ Montreal Neurological Institute, McGill University, Montréal, QC Canada; ^4^ McConnell Brain Imaging Centre ‐ McGill University, Montreal, QC Canada; ^5^ McConnell Brain Imaging Centre, Montreal Neurological Institute and Hospital, McGill University, Montreal, QC Canada; ^6^ Montreal Neurological Institute, McGill University, Montreal, QC Canada; ^7^ University of Gothenburg, Mölndal, Gothenburg Sweden; ^8^ Department of Psychiatry and Neurochemistry, Institute of Neuroscience and Physiology, The Sahlgrenska Academy, University of Gothenburg, Mölndal, Gothenburg Sweden; ^9^ The Sahlgrenska Academy at the University of Gothenburg, Mölndal Sweden; ^10^ Department of Psychiatry and Neurochemistry, Institute of Neuroscience and Physiology, University of Gothenburg, Mölndal Sweden; ^11^ Department of Psychiatry, McGill University, Montréal, QC Canada; ^12^ Centre for Studies on Prevention of Alzheimer's disease (StoP‐AD Centre), Montreal, QC Canada; ^13^ Department of Medicine, McGill University, Montréal, QC Canada; ^14^ McGill Centre for Integrative Neuroscience, McGill University, Montreal, QC Canada; ^15^ The Douglas Research Center, Montreal, QC Canada; ^16^ StoP‐AD Centre, Douglas Mental Health Institute Research Centre, Montreal, QC Canada; ^17^ Centre for Studies on Prevention of Alzheimer's disease (StoP‐AD Centre), Douglas Mental Health Institute, Montreal, QC Canada

## Abstract

**Background:**

For medical purposes, amyloid‐beta (Aβ) and tau biomarkers are typically dichotomized into positive (+) and negative (‐) status to define individuals with Alzheimer’s disease (AD) pathology. Nevertheless, such AD proteinopathies start accumulating years before reaching clinically‐defined abnormality thresholds. We examined longitudinal change in PET Aβ and tau in cognitively unimpaired (CU) individuals; then we explored their baseline plasma levels and demographic characteristics. Finally, we assessed the association between plasma/demographics and annual rate of change (aRC) of AD pathology.

**Method:**

We included 110 CU participants from the PREVENT‐AD cohort who underwent longitudinal Aβ and tau PET scans (mean time between scans = 4.33 years, 0.44 SD). We created four accumulator groups: 1) a non‐accumulators group, 2) an Aβ‐only group which included individuals classified as Aβ+ at baseline (SUVr threshold of 1.25, 18 CL) plus those with an Aβ aRC > 2.19 CL, 3) a tau‐only group which included individuals classified as tau+ at baseline (2 SDs above the mean of young participants, SUVr=1.23) plus those with a temporal meta‐ROI aRC > 0.022 (a GMM‐derived cut‐point), and 4) an Aβ & tau accumulator group. We then compared demographic characteristics and baseline plasma biomarker levels of Aβ42/40, GFAP, NfL, pTau181 and pTau217 across the four groups. Finally, we fitted linear regression models to predict Aβ/tau aRC as continuous variables from demographics or baseline plasma, including age and sex as covariates.

**Results:**

Aβ accumulator groups included a higher proportion of APOE4 carriers (Table 1). Compared to non‐accumulators or tau‐only accumulators, Aβ‐only accumulators and Aβ & tau accumulators showed higher baseline plasma levels of NfL and GFAP. When aRC was treated as a continuous value, APOE4 status and age were positively associated with higher rate of Aβ accumulation. Finally, higher baseline GFAP levels were associated with faster accumulation of both Aβ and tau in the brain

**Conclusions:**

APOE4 status, increased age and plasma GFAP levels were closely related to faster Aβ aRC. Higher plasma GFAP levels were further associated with faster tau aRC. Therefore, GFAP might be an indicator of AD pathophysiological processes.